# Utilization of Intraoperative TEE to Assess Supraventricular Tachycardia-Inducing Right-Sided Cardiac Compression by the Liver, Post-Liver-Transplantation Status

**DOI:** 10.1155/2015/136595

**Published:** 2015-03-12

**Authors:** W. David Stoll, William R. Hand, Vinayak S. Rohan, Parker M. Gaddy, Scott T. Reeves, Kenneth D. Chavin

**Affiliations:** ^1^Department of Anesthesia and Perioperative Medicine, Medical University of South Carolina, Charleston, SC 29425, USA; ^2^Department of Surgery, Division of Transplant Surgery, Medical University of South Carolina, Charleston, SC 29425, USA; ^3^Department of Surgery, Division of Transplant Surgery, Microbiology and Immunology, Medical University of South Carolina, Charleston, SC 29425, USA

## Abstract

This unique and interesting case report involves a patient who recently underwent a combined liver and kidney transplant (due to autosomal dominant polycystic kidney disease) and subsequently suffered from episodes of supraventricular tachycardia (SVT) secondary to the new liver graft compressing the right atrium and ventricle. After this was diagnosed, the patient underwent operative plication of the right hemidiaphragm. Intraoperative transesophageal echocardiography was used to demonstrate cardiac compression from the liver and demonstrate resolution of compression after plication of the hemidiaphragm.

## 1. Case Description

**Figure 1:**
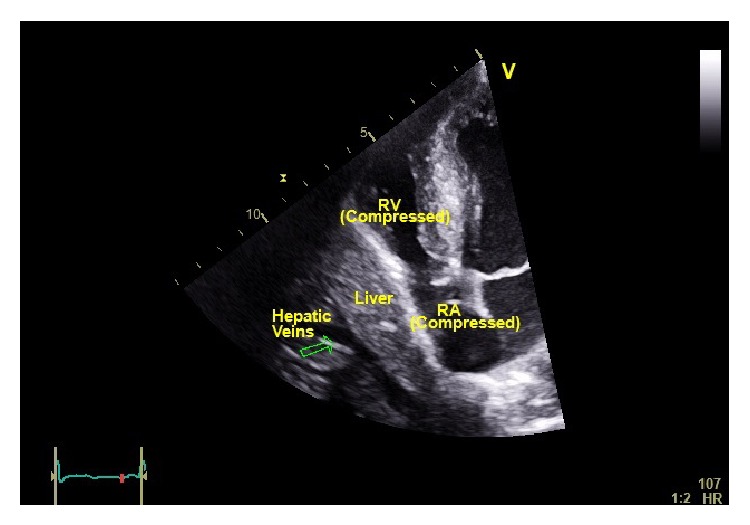
Transthoracic apical four-chamber image obtained after transplant but prior to the diphragm plication. Hepatic compression of the atrium and ventricle is evident here. RV: right ventricle, RA: right atrium.

A 55-year-old African American male was taken to the operating room for diaphragmatic plication. He had successfully undergone a combined liver and kidney transplant two weeks prior to autosomal dominant polycystic disease. Following transplantation, the patient developed multiple recurrent episodes of supraventricular tachycardia (SVT). The dysrhythmia was confirmed by numerous bedside ECGs and extensive testing ensued to discern the etiology. Pertinent subsequent investigations of the SVT included plain film chest X-rays, transthoracic echocardiogram (TTE), right heart catheterization (RHC), and pulmonary function testing (PFT). The chest radiograph revealed an elevated right-sided hemidiaphragm while TTE demonstrated a small right atrium (RA) that appeared compressed along with the right ventricle (RV) (Figure 1). Subsequent right heart catheterization indicated a small right atrium with compression that appeared to compromise flow. Finally, pulmonary function testing demonstrated a restrictive pattern. This testing, in combination with laboratory testing and patient history, helped to rule out other causes such as myocarditis, pneumonia, drugs/toxins (e.g., cocaine), heart failure, endocrine (e.g., thyroid), and pulmonary emboli.

Based upon these findings, the surgical plan was to plicate the diaphragm to prevent compression of the heart (and thus prevent episodes of SVT). In addition to cardiac evaluation, we felt that an intraoperative transesophageal echocardiogram (TEE) would be helpful in further evaluating the elevated right hemidiaphragm. After induction of general endotracheal anesthesia, the patient developed SVT while attempting to roll him in the left lateral decubitus position. Intravenous adenosine (6 mg IV push × 1) was given with conversion to sinus rhythm. Due to this positional instability, the procedure was performed in the supine position and a TEE probe was placed without incident. A standard American Society of Echocardiography comprehensive examination was performed.

**Figure 2:**
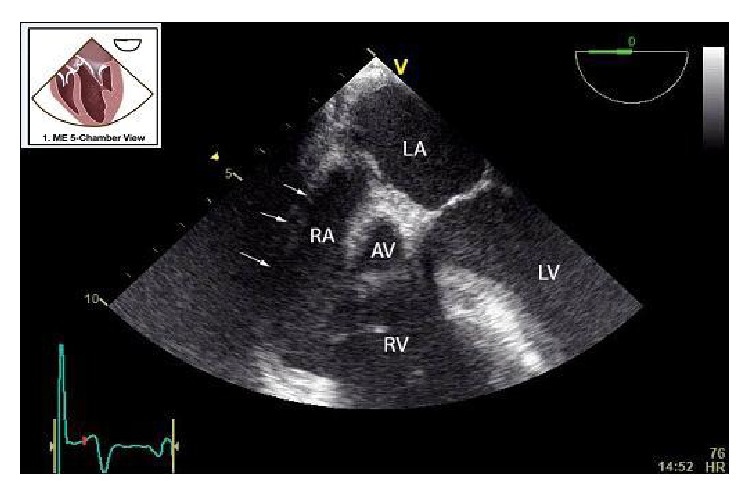
Midesophageal (ME) five-chamber view. Arrows delineate the lateral wall of the right atrium (RA) (see Supplemental Digital Content 1 for the associated video in the Supplementary Material available online at http://dx.doi.org/10.1155/2015/136595). A mass of echogenic pattern consistent with hepatic tissue is visualized adjacent to and compressing the RA. Cartoon inset = ME 5-chamber view adapted from ASE and SCA Guidelines and Standards [7]. AV: aortic valve, LA: left atrium, LV: left ventricle, and RV: right ventricle.

**Figure 3:**
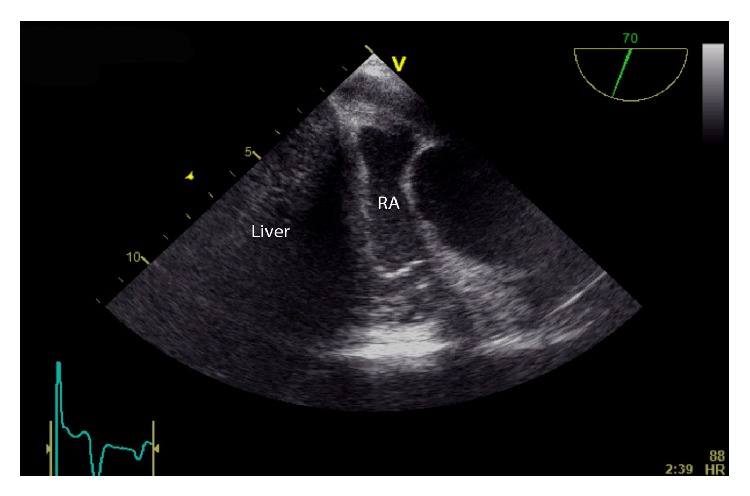
ME image obtained at 70 degrees to better visualize the liver compressing the right atrium (RA) (see associated Supplemental Digital Content 2).

The surgery proceeded without incident after the initial run of SVT during patient positioning. Once the surgeons entered the thorax, it was obvious that the right hemidiaphragm was severely displaced into the thorax thus allowing for position-related compression of the heart. Compression of the right atrium by the transplanted liver is demonstrated (Figures 1–3, Videos 1 and 2). TEE was useful in confirming the etiology and guiding the surgeons through a successful right-sided diaphragmatic plication.

## 2. Discussion

Starting with the midesophageal (ME) four-chamber view, the right atrium was compressed even with the patient in the supine position. Ventricular compression as seen during the preop TTE was not appreciated most likely due to the TTE being performed in the left lateral decubitus position. In the ME five-chamber view (Video 1), an echogenic mass appeared to be compressing the right atrium. By turning the omniplane angle to 70 degrees, the RA compression was more evident, and the echogenic mass was clearly the diaphragm and liver (Video 2). After investigating the compression in two views, we returned to the ME five-chamber view. At this point, the surgeons retracted the liver off of the diaphragm and the myocardial compression was significantly improved (Video 3). Unfortunately, postplication TEE images were not available. Postoperative TTE follow-up confirmed complete resolution of any RA or RV compression by the transplanted liver (Video 4 = apical 4-chamber view). Additional postplication testing included pulmonary function testing (PFT) with no significant decrement in function (Pre FEV1/FVC 70% versus 72% Post and Pre FVC 1.84 L versus 2.03 L Post).

This report describes how an unusual set of circumstances led to a symptomatic cardiac dysrhythmia in patient status after combined liver-kidney transplantation. Intraoperative TEE assisted in confirming the pathophysiologic mechanism presumed to cause SVT. Additionally, TEE confirmed that the right diaphragmatic plication was successful in eliminating compression of the right atrium by the transplanted liver prior to leaving the operating room. The authors are pleased to have assisted in confirming the diagnosis and facilitating the treatment of this unusual postoperative complication. Numerous reports exist confirming the obvious utility of TEE in assessment of cardiac masses. Most reports describe intracardiac masses such as thrombi, lipomas, and myxomas as well as masses of unknown etiology [1]. Fewer reports exist on the echocardiographic assessment and surgical guidance on the resection of intracardiac masses [2, 3]. Only a handful of reports exist describing cardiac compression from external masses [4–6]. To our knowledge, however, this is the first report of symptomatic myocardial compression by the liver in which surgical resolution was guided and confirmed via real-time TEE imaging. We believe this report to be a useful description and addition to the current paucity of literature describing extracardiac masses.

## Supplementary Material

The supplemental digital material for this case report is contains four separate echocardiographic videos. Video 1 is a mid-esophageal five chamber view and the arrow delineates the lateral wall of the right atri-um. A mass of echogenic pattern consistent with hepatic tissue is visualized adjacent to, and compressing the right atrium. Video 2 is a mid-esophageal four chamber view that has been omniplaned to 70 degrees to better visualize the right atrium. Right atrial compression is evident. The next video (Video 3) is again the mid-esophageal five chamber view but it was acquired while the surgeons retracted the liver. It is evident that compression of right atrium is significantly improved by the surgical maneuver. Lastly, Video 4 is an apical four chamber transthoracic echocardiography loop that was captured post-plication. Compression of the right atrium and right ventricle is no longer present. Key: AV: Aortic Valve, LA: Left atrium, LV: Left Ventricle, RA: Right Atrium, RV: Right ventricle. TV: Tricuspid Valve.
